# Characterization of *Bacillus anthracis* replication and persistence on environmental substrates associated with wildlife anthrax outbreaks

**DOI:** 10.1371/journal.pone.0274645

**Published:** 2022-09-21

**Authors:** Treenate Jiranantasak, Jamie S. Benn, Morgan C. Metrailer, Samantha J. Sawyer, Madison Q. Burns, Andrew P. Bluhm, Jason K. Blackburn, Michael H. Norris

**Affiliations:** 1 Department of Geography, Spatial Epidemiology & Ecology Research Laboratory, University of Florida, Gainesville, Florida, United States of America; 2 Emerging Pathogens Institute, University of Florida, Gainesville, Florida, United States of America; US Geological Survey, UNITED STATES

## Abstract

Anthrax is a zoonosis caused by the environmentally maintained, spore-forming bacterium *Bacillus anthracis*, affecting humans, livestock, and wildlife nearly worldwide. Bacterial spores are ingested, inhaled, and may be mechanically transmitted by biting insects or injection as occurs during heroin-associated human cases. Herbivorous hoofstock are very susceptible to anthrax. When these hosts die of anthrax, a localized infectious zone (LIZ) forms in the area surrounding the carcass as it is scavenged and decomposes, where viable populations of vegetative *B*. *anthracis* and spores contaminate the environment. In many settings, necrophagous flies contaminate the outer carcass, surrounding soils, and vegetation with viable pathogen while scavenging. Field observations in Texas have confirmed this process and identified primary browse species (e.g., persimmon) are contaminated. However, there are limited data available on *B*. *anthracis* survival on environmental substrates immediately following host death at a LIZ. Toward this, we simulated fly contamination by inoculating live-attenuated, fully virulent laboratory-adapted, and fully virulent wild *B*. *anthracis* strains on untreated leaves and rocks for 2, 5, and 7 days. At each time point after inoculation, the number of vegetative cells and spores were determined. Sporulation rates were extracted from these different time points to enable comparison of sporulation speeds between *B*. *anthracis* strains with different natural histories. We found all *B*. *anthracis* strains used in this study could multiply for 2 or more days post inoculation and persist on leaves and rocks for at least seven days with variation by strain. We found differences in sporulation rates between laboratory-adapted strains and wild isolates, with the live-attenuated strain sporulating fastest, followed by the wild isolates, then laboratory-adapted virulent strains. Extrapolating our wild strain lab results to potential contamination, a single blow fly may contaminate leaves with up to 8.62 x 10^5^ spores per day and a single carcass may host thousands of flies. Replication outside of the carcass and rapid sporulation confirms the LIZ extends beyond the carcass for several days after formation and supports the necrophagous fly transmission pathway for amplifying cases during an outbreak. We note caution must be taken when extrapolating replication and sporulation rates from live-attenuated and laboratory-adapted strains of *B*. *anthracis*.

## Introduction

Anthrax is an underreported zoonosis often resulting in large epizootics in livestock and wildlife with spillover to humans most often associated with animal slaughter or meat handling (livestock or bush meat[1]) [2]. *Bacillus anthracis*, the causative agent of anthrax, is a Gram-positive bacterium naturally occurring in the environment worldwide where it forms resilient, dormant spores capable of surviving for extended periods of time [3, 4]. It is generally understood that persistence in the environment allows outbreaks in subsequent years [2, 5], but there is a notable gap in knowledge related to case amplification during an outbreak. In various enzootic anthrax areas, patterns in wildlife anthrax outbreaks have been linked to seasonality, grazing animals [6, 7] and biting fly transmission [8–10], but the exact mechanisms of transmission during an outbreak remain undefined for the browsing species most frequently affected in North America.

Herbivorous hoofstock are highly susceptible to anthrax, and in North America most outbreaks associated with wildlife affect wood bison (*Bison bison athabascae*), and plains bison (*Bison bison bison*) [7, 11], elk (*Cervus canadensis*), and white-tailed deer (*Odocoileus virginianus*) [12]. In the United States, anthrax outbreaks in wildlife occur most frequently in Texas where there are large ranching operations with numerous susceptible native and nonnative (exotic) Bovidae (antelope) and Cervidae (deer) species [13]. Animals succumbing to anthrax typically have high fevers [14] and we note anecdotally that many carcasses found in West Texas scrub are in cooler areas with water sources and dense vegetation for shading; these areas represent a complicated mixture of soil, organic material, and rocks. The infected carcass serves as a source of viable *B*. *anthracis* cells on these resource rich landscapes, creating a localized infectious zone (LIZ) with the potential to initiate a future outbreak [6], but fly transmission pathways can play a more immediate role in anthrax exposure during an active outbreak [11, 13, 15].

There are multiple routes of insect-mediated exposure in this complex disease transmission system [16]. For example, necrophagous flies are central to the case multiplier hypothesis which suggests that flies could increase the severity of an epizootic by increasing contact opportunities between *B*. *anthracis* contaminated fly droplets and naïve animals [15]. After an animal dies, mature necrophagous flies colonize and feed on carcasses in high numbers while laying eggs [17, 18]. If the animal died from anthrax, necrophagous flies contaminate the areas immediately surrounding the carcass with vegetative and sporulated *B*. *anthracis*, spreading infectious materials onto nearby vegetation including leaves, grasses, and rocks via regurgitation and defecation [19]. This results in expansion of the LIZ and potential for increased case numbers when naïve animals are exposed to this contaminated area [5, 7, 11]. Early research has confirmed the presence of anthrax bacilli in fly emesis and feces [20, 21], and prior research by our laboratory verified flesh-eating flies collected directly off of anthrax-positive deer carcasses were carrying viable *B*. *anthracis* [22]. Flies have also been confirmed to contaminate vegetation surrounding a carcass [19] which expands the LIZ to forbs and browse species, such as Texas persimmon (*Diospyros texana*), a primary source of protein in the summer months for white-tailed deer [11]. This necrophagous transmission pathway has been illustrated previously [11]. Additional data from Etosha National Park (ENP), Namibia, confirmed blow flies had 4.0 x 10^3^ organisms, enough for parenteral LD_50_ in impala (*Aepyceros melampus*), and contamination around carcasses, though browse plant contamination was not established [23]. Such contaminated browse, which has been confirmed in Texas, can then expose other animals to *B*. *anthracis* via ingestion and continue the outbreak cycle [13, 19, 24, 25].

The research described above thus far supports insect-mediated *B*. *anthracis* transmission via environmental contamination as well as the mechanical spread of viable cells from contaminated mouthparts, but it has yet to be thoroughly investigated. Preliminary estimates of LIZ sizes have accounted for contamination immediately surrounding a carcass and in the soil [6, 7], a field study in Texas confirmed flies collected from leaves 3–5 meters away from an anthrax carcass were positive for *B*. *anthracis* by culture and PCR [11], which expands the diameter of the LIZ outward several meters and vertically from the ground and carcass. Although anthrax spore stability in the soil may allow for outbreaks years apart, prior results suggest anthrax persistence in the LIZ during an outbreak may be spatiotemporally constrained as a previous field study did not recover viable bacteria from visually contaminated leaf samples collected near a carcass that was at least 21 days old [11].

Understanding the dynamics of *B*. *anthracis* vegetative cell and spore persistence on environmental substrates together with insect-mediated bacterial transport will expand the limited knowledge of fly transmission pathways during outbreaks, particularly in browse dominated anthrax systems [5]. We hypothesize that *B*. *anthracis* containing fly droplets can expand the LIZ to produce a large sphere of contamination surrounding an anthrax carcass after death, thereby increasing exposure risk and hazard of infection to animals feeding or otherwise contacting this material. Towards answering this hypothesis, we sought to understand the number of bacteria that remain viable following simulated insect deposition of *B*. *anthracis* on environmental substrates. We quantified viable *B*. *anthracis* vegetative cell and spore recoveries from leaves and rocks to better understand the risk associated with LIZs and necrophagous fly transmission following host death. In addition, we examined differences in replication, persistence, and sporulation on environmental substrates between laboratory-adapted strains and wild strains recovered from Texas outbreaks, which may be an important consideration when developing representative laboratory models.

## Materials and methods

### Outbreak carcass, fly identification, and environmental substrate imaging

All animal carcasses were part of private wildlife management operations or properties and found in the normal course of landscape surveillance during the 2019 Texas anthrax outbreak that occurred in the West Texas anthrax triangle [26], a region that experiences enzootic anthrax [27]. The University of Florida (Gainesville, FL, USA) Institutional Animal Care and Use Committee (IACUC) does not require approval or waivers for imaging or sampling of dead animals found during landscape transects. There were no interactions with live animals in the course of this work.

**Fig 1:**
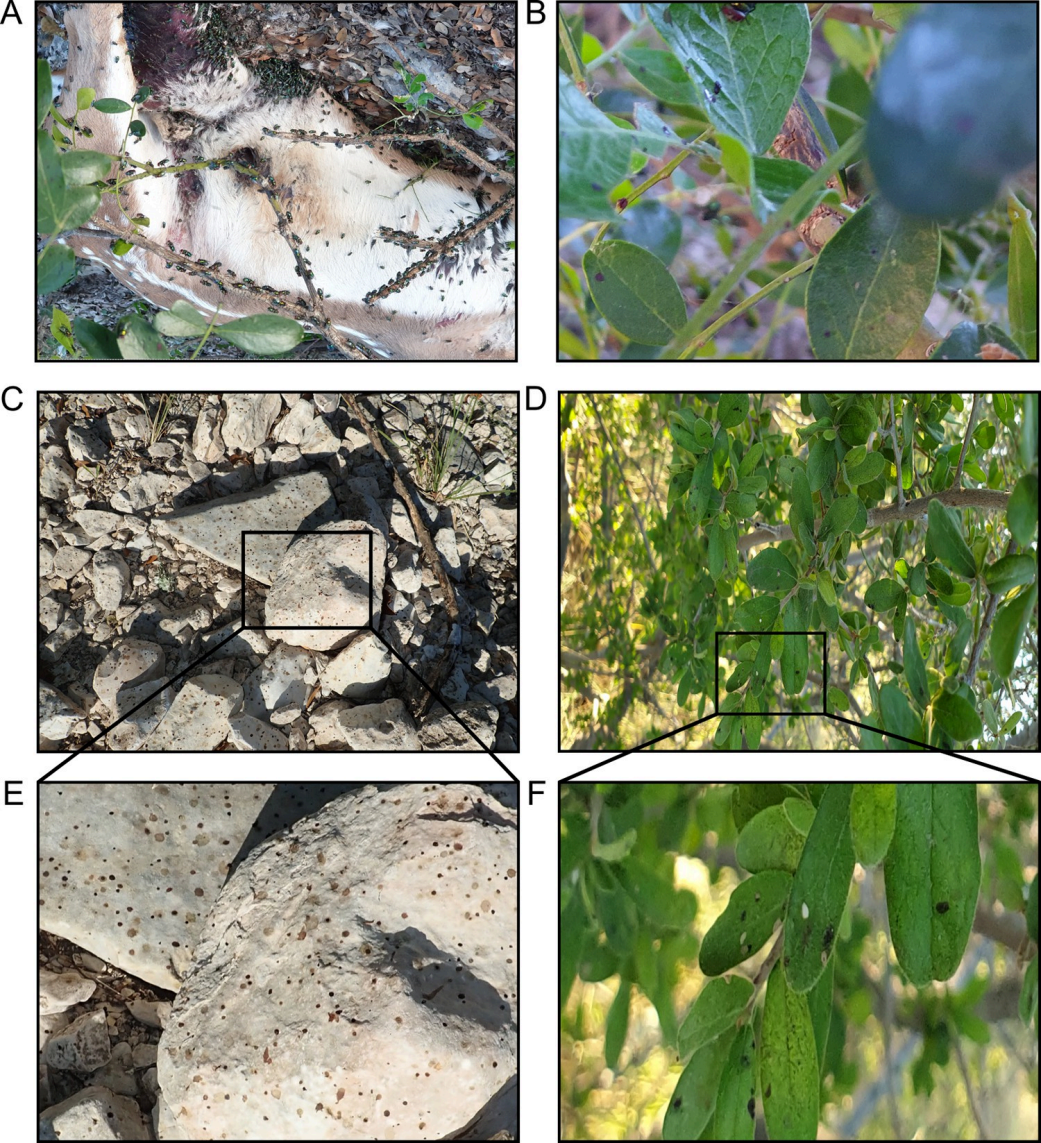
Environmental contamination in the localized infectious zone (LIZ). Pictures captured during an anthrax outbreak associated with wildlife in West Texas in 2019 representing the high number of flies feeding on a carcass (A), flies contaminating leaves (B), fly deposits on rocks (C), and fly deposits on leaves near an anthrax carcass (D) that is typical of anthrax outbreaks in this area. Insets magnify fly deposits on rocks (E) and leaves (F).

*Chrysomya rufifacies* (M.) (Diptera: Calliphoridae) and Sarcohphagidae were identified as the likely taxa from deer known to have perished from anthrax in West Texas in 2019 (Fig 1A). Taxa were determined based on the known species documented in West Texas during the summer when the images were taken [28] and using gestalt characters from images of flies from dorsal and lateral views at the site. For example, majority of flies documented had both green abdomen and thorax, a robust body shape with darker banding on the abdomen, characters commonly associated with the *Chrysomya* spp. *Chrysomya rufifacies* and *Chrysomya megacephala* (F.) are possible candidates geographically, however *C*. *rufifacies* has been documented more in the summer months in Texas [28], and has white gena [29] which is clearly photographed at the site, unlike *C*. *megacephala* that has yellow gena [29] with no documentation of the flies in photographs having this character.

All laboratory work was conducted under approved Institutional Biosafety Committee (IBC) protocols at the University of Florida. To correlate volumes of liquid with observed emesis diameters, various volumes of 6X gel loading dye (New England Biolabs, Ipswich, MA, USA) were spotted onto petri dishes in triplicate and allowed to dry overnight. Images were captured on a BioRad Gel Doc XR+ Gel Documentation System (Bio-Rad Laboratories, Hercules, CA, USA). Spot diameter was determined by measurement calibration with a ruler and using Fiji image processing software (NIH) to measure the spot diameter. The same approach was used to measure fly emesis diameters from our repository of rock and leaf samples collected at different sites during the 2019 Texas anthrax outbreak. Subsamples of the same rock and leaf samples had viable pXO1 and pXO2 positive *B*. *anthracis* spores on their surfaces. These bacteria were genome sequenced and positively identified as *B*. *anthracis* (in preparation).

### Bacterial strains

All strains used in this study are archived in the Martin E. Hugh-Jones *Bacillus anthracis* Collection at the University of Florida. Selected *B*. *anthracis* were handled in a CDC/USDA registered and inspected BSL3 facility according to the practices and procedures recommended by the Biosafety in Microbiological and Biomedical Laboratories 6^th^ edition [30]. We used four fully virulent strains of *B*. *anthracis* previously analyzed for their proteomic profiles and sporulation abilities [31, 32]. Two strains, an Ames-like strain of the A.Br. Ames lineage (UF01106) and Vollum-like of the A.Br.Vollum lineage (UF01103), were wild isolates that infected white-tailed deer (*Odocoileus virginianus*) from geographically distinct outbreaks on West Texas ranches in 2009 and had been cultured no more than three times, while “lab” strains, Ames (UF00738; alternate identifier A0462) and Vollum (UF00980; alternate identifier A0084 or Vollum 1), had been cultured through more than 100 passages. The fifth strain used was Sterne strain (34F2; Colorado Serum Company; Denver, CO, USA), a live-attenuated strain lacking the pXO2 plasmid commonly used in veterinary vaccines to protect against anthrax. This strain often serves as a biosafe (BSL-2) proxy strain for *B*. *anthracis* experiments. The presence or absence of chromosomal, pXO1 and pXO2 markers in these strains was confirmed by qPCR as previously described [11, 33].

### Environmental substrates

Rock samples were natural gravel collected from a single area in northern Florida as representative of a natural abiotic surface. No permit was required for the collection of rock samples from private land as permission was given by the homeowner. Leaf samples were gathered from a Southern live oak tree (*Quercus virginiana*) in Gainesville, FL, USA as a representative LIZ adjacent biological surface with leaves of a similar length (2–15 cm) and width (1–5 cm) compared to Texas persimmon (*Diospyros texana*) [34]. Environmental samples were not decontaminated prior to use in experimental trials to avoid potential alteration of physical and chemical characteristics of leaves and rocks.

### Environmental substrate inoculation and recovery

Fully virulent strains of *B*. *anthracis* were streaked onto brain-heart infusion (BHI) agar (Difco, BD Life Sciences, Sparks, MD, USA) and incubated at 35°C overnight. Isolated colonies from the incubated plates were used to inoculate 3 ml of BHI broth (Difco, BD Life Sciences, Sparks, MD, USA) in sterile 0.22 μm ventilated cap 15 ml tubes (CELLTREAT Scientific Products, Pepperell, MA, USA). For the Sterne strain, BHI broth was inoculated directly from the frozen glycerol stock, then all inoculated broths were shaken at 220 rpm at 35°C ± 2°C overnight. Resulting cultures were diluted in sterile BHI broth and the original OD_600_ was determined for each inoculation broth using a Thermo Scientific GENESYS20 spectrophotometer (Thermo Fisher Scientific, Waltham, MA, USA). All cultures were diluted to an OD_600_ of 1 using sterile BHI broth and used to inoculate environmental substrates. Leaf and rock samples were spotted in duplicate with a single 20 μl droplet of the culture. Inoculated samples were incubated at 30°C until being processed at 2-, 5- and 7-days post inoculation (dpi). Wildlife interaction with anthrax carcasses can vary during this time, with zebra grazing at carcass sites being highly probable following animal death while wildebeest interaction highly improbable [6]. Female and male bison interact with carcass sites at differing probability depending on the season and species [7]. Insect interaction with the animal carcass is greatest during this time-period after death and was the highest probability of insect involvement so our experiments focused on understanding bacterial characteristics over the first 7 days after deposition [17, 18]. The starter culture and additional leaf and rock samples were inoculated and processed on the same day to determine inoculation bacterial titers and assess recovery at Day 0 (D0).

At each timepoint, inoculated leaf and rock samples stored in 50 ml conical tubes were suspended in 5 ml of PBS with 0.05% Tween-20 (PBST) with approximately 1 ml of acid-washed 425–600 μm glass beads (Sigma-Aldrich, St. Louis, MO, USA). Samples were bead beat for 5 min using a vortexer and centrifuged at 250 x g for 2 min. Supernatants were then transferred to sterile sample tubes for further processing. One aliquot was serially diluted in PBST, plated on tryptic soy agar (TSA) (Research Products International, Mount Prospect, IL, USA), and incubated at 37°C to determine total colony forming units (CFU). Another 300 μl aliquot of each supernatant was added to 700 μl of 100% ethanol to reach a final concentration of 70% ethanol to kill any vegetative bacteria and allow enumeration of spores. These aliquots were incubated at room temperature for 1 h with 1 min of vortexing at 15-min intervals to ensure homogenous ethanol exposure throughout the solution. Ethanol treated samples were then serially diluted in PBST, plated on TSA, and incubated at 37°C overnight to count bacterial CFU. CFU were multiplied by the dilution factor to obtain the number of spores. The number of vegetative cells were derived by subtracting spores from the total CFU. The characteristic *B*. *anthracis* colony morphology was used to confirm the recovery of vegetative cells and spores were from the target organism and not native rock or leave flora. This experiment was carried out two independent times with two biological replicates each time for each strain.

### Statistical analysis

On leaves and rocks, CFU and spores per sample were calculated by multiplying colony number by the dilution factor, then spores were subtracted from corresponding CFU to calculate the number of vegetative cells for each replicate. Mean and standard error of the mean (SEM) of resulting bacterial quantities for total cells, vegetative cells and spores were graphed on a log scale. Mean and SEM of total cells and spores recovered for grouped fully virulent laboratory (high passage) and wild (low passage) strains were graphed on a log scale. Sporulation rate (spores/day) was calculated from the slope of the lines between days for each timepoint. The mean and SEM for each strain and for grouped fully virulent laboratory (high passage) and wild (low passage) strains were graphed on a linear scale.

To determine sample distribution, Shapiro-Wilk tests were performed. Many of these data were not normally distributed. Therefore, we used non-parametric tests to determine significance for all experiments. A Mann-Whitney U test was used to determine significance between surface types and between grouped fully virulent laboratory (high passage) and wild (low passage) strains on D2, D5, and D7. A Kruskal-Wallis test with Dunn’s multiple comparisons test was used to determine significance between days for each strain, as well as between strains at each timepoint. All statistical analyses and graph production were performed in Graph Pad PRISM software (GraphPad Software, San Diego, California, USA) with significance determined at *ρ* < 0.05.

### Extrapolation of possible anthrax contamination levels caused by necrophagous flies

Data from Rivers and McGregor [35] determining the variation in volume and morphometrics of blood stains generated by flies (fly spots) from *C*. *rufifacies* and *Sarcophaga bullata* (P.) (Diptera: Sarcophagidae) provided different resource types (fluids: human blood (3 ml), bovine blood (3 ml), and tissues: bovine liver (50 g), and a mouse carcass (~25 g)) were used to extrapolate fly spot volumes to determine potential spread of *Bacillus* CFU and spores as determined in this laboratory study by an individual fly.

Surface area calculations were generated for the 30 cm x 30 cm x 30 cm BugDorm-1^®^ (Taiwan) fly cages and the 110 mm Whatman (United Kingdom) No. 4 Qualitative Disc Filter Paper used in the experiment to estimate the total surface area 100 flies had access to over a 24-hour period with a 15:9 L:D cycle [35]. With the assumption that flies distributed emesis on all available surfaces equally, the volume of

$$\frac{\left((\begin{array}{c} Blood\;on\;Filter\;Paper\;(\mu L)\\ \frac{x\;Cube\;Surface\;Area\;(mm^{2})}{Filter\;Paper\;Surface\;Area\;(mm^{2})} \end{array})+Blood\;on\;Filter\;Paper\;(\mu L)\right)}{100\;Flies}=Average\;Volume\;(\mu L)\;per\;Fly$$

fluid (μL) calculated from [35] was used to estimate the proportional value of emesis on the cube surface to summate the total volume of emesis generated within the 24 hour period for the fly population. The total volume was then divided by 100 to determine the average amount of emesis each fly generated overall. The same steps were taken to determine the number of spots generated by each fly within the 24 hours.

Blood spots were categorized as round, asymmetric, translocation, and tarsal tracks based on shape and size with the percentage of spot type provided [35]. To determine the number of spots per spot type per fly, the number of spot type per filter paper was derived from the percentage of spot types and total number of spots. Then, the number of fly spots of a given type were used to determine the proportional number of spots in the cube, assuming the filter paper was representative of all available surfaces. The total number of spots of a given type from the filter paper and cube were then divided by the 100 flies in the cage.


$$\left((\frac{\left((\frac{Percent\;Spot\;Type\;x\;Number\;of}{100})\;x\;Cube\;Surface\;Area\right)}{\frac{Filter\;Paper\;Surface\;Area\;(mm^{2})}{100\;Flies}})+Number\;of\;Spots\;on\right)=Average\;Number\;of\;Spot\;Type\;per\;Fly$$

To determine the volume of each spot type, two steps were taken. First, scaled images from [35] were used to compare the spot sizes generated by each spot type from *C*. *rufifacies* and *S*. *bullata* that consumed human blood. Using this information, estimations of volume per spot type were calculated by taking the percentage of the total spot volume per each spot type and dividing by the number of spots per spot type per fly in a 24-hour period. These values were then used to determine potential CFU and spore contamination levels possible from both wild type *B*. *anthracis* strains used in this study (UF01103 and UF01106) by multiplying CFU and spores measured at Day 0 (D0) by the volumes of emesis spread in a 24 h period.

## Results

### Outbreak carcass, environmental substrate imaging, and droplet volume determination

**Fig 2:**
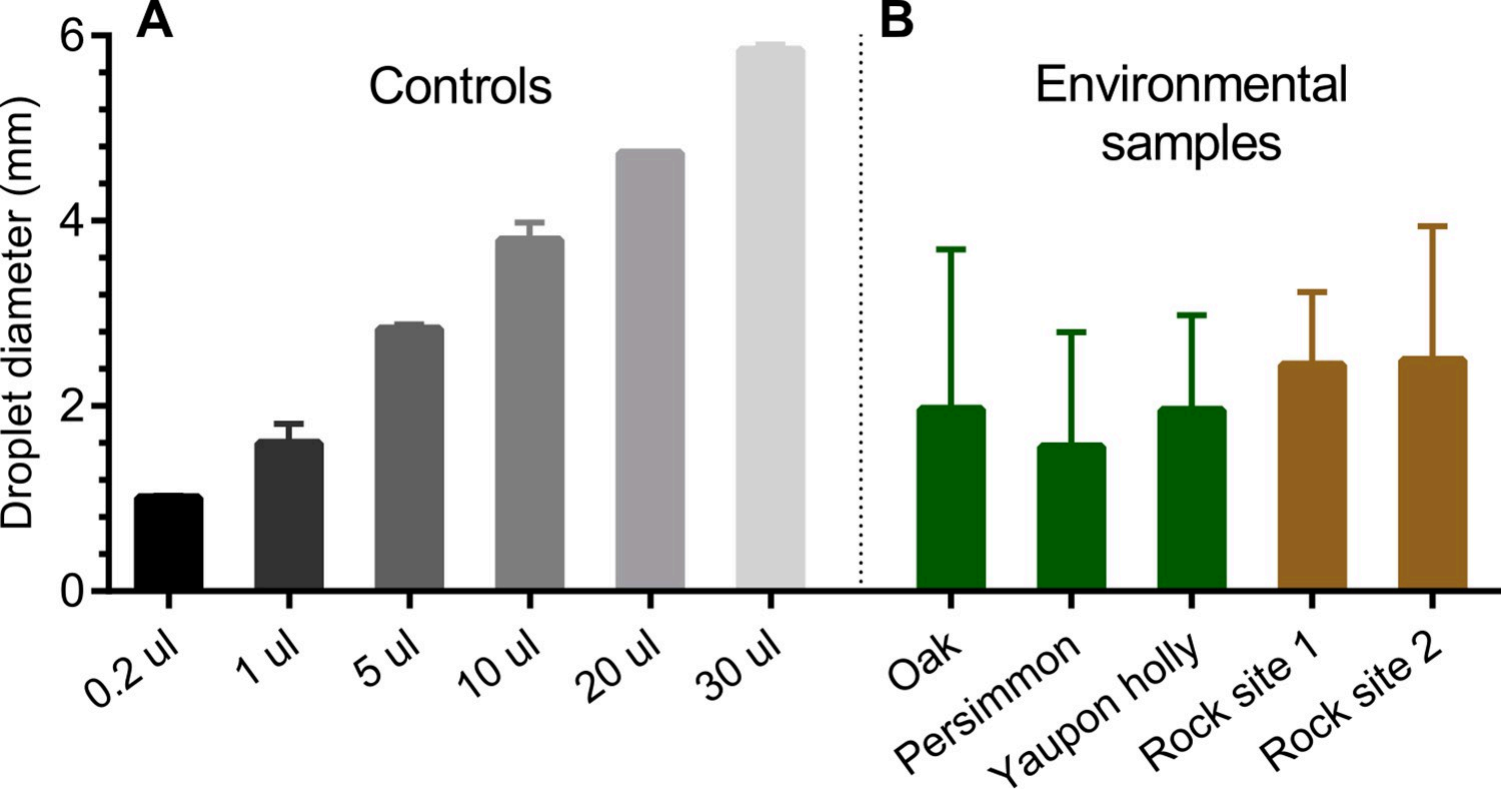
A comparison of diameters between laboratory experiments and samples collected from the outbreak in Texas. (A) Averages of droplet volume spotted onto petri dishes in controlled experiments. (B) Averages of fly droplets and emesis on leaves (12 spots from 7 yaupon leaves, 20 spots from 9 oak leaves and 6 spots from3 persimmon leaves) and rocks (10 spots from 2 rocks at site one and 10 spots from 3 rocks at site 2) collected during the 2019 anthrax outbreak in Texas where viable *B*. *anthracis* spores were isolated.

Animals recently dead from anthrax are found across the landscape during outbreaks of anthrax. The spore contaminated carcasses become important nutrient sources for necrophagous fly replication, exemplified in Fig 1A. Emesis deposited on the surrounding environment of leaves and rocks as the flies feed on anthrax-infected carcasses (Fig 1B–1F), spreads the zone of contamination beyond the carcass surface. A preliminary analysis of emesis diameters found on leaves and rocks collected from a past anthrax outbreak in Texas was used to correlate emesis diameters to droplet volumes in the controlled laboratory experiments (Fig 2A and 2B). Outbreak contaminated leaf and rock materials with viable spore isolations had measurable fly excretion diameters as large as 4 mm (Fig 2A). Based on these observations and initial experiments showing high variability with 10 μl spots, a volume of 20 μl was taken as the droplet volume that encompassed all diameters of fly emesis measured on *B*. *anthracis* positive rocks and leaves.

### Viability and multiplication of *Bacillus anthracis* vegetative cells and spores on environmental substrates

**Fig 3:**
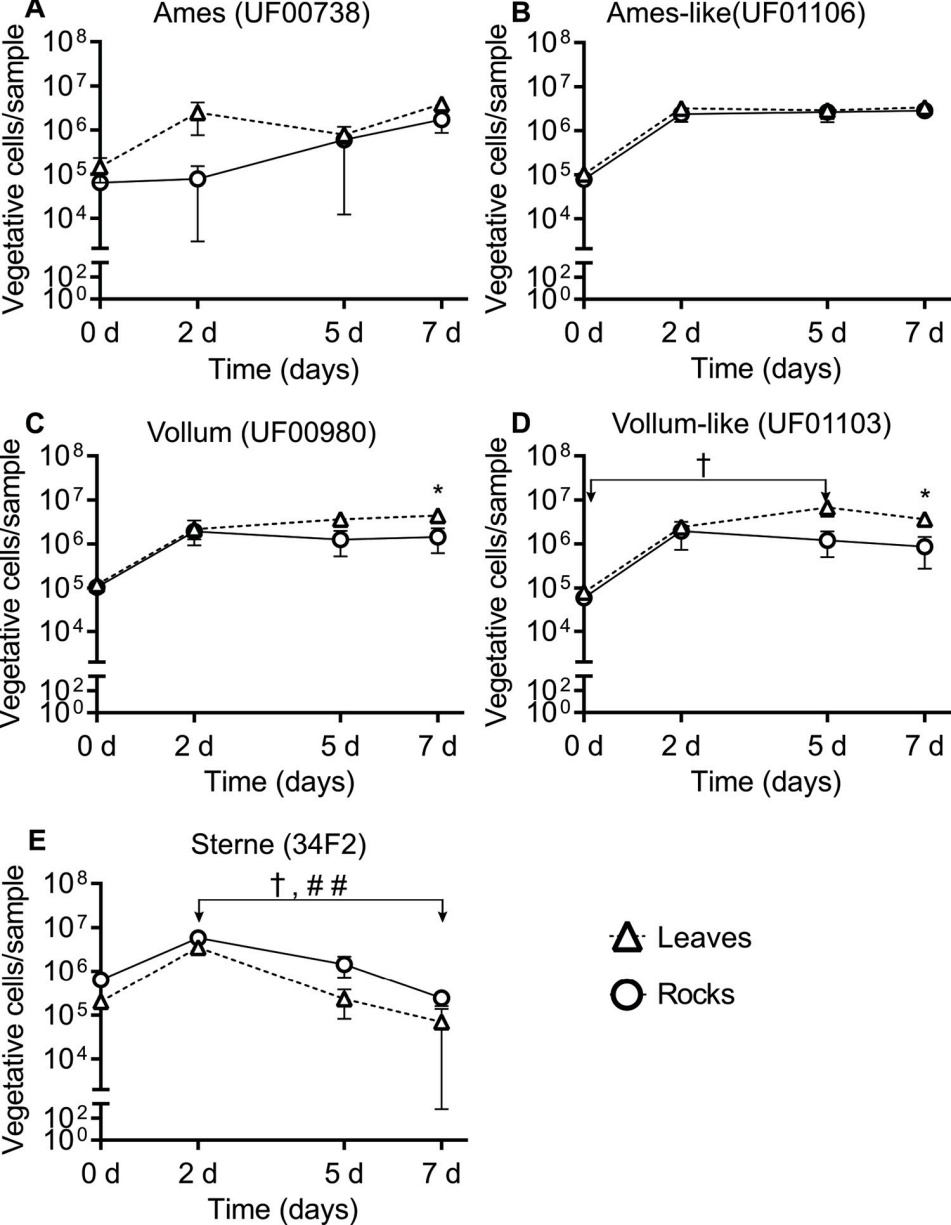
*Bacillus anthracis* vegetative cell viability on leaf and rock surfaces over a 7-day period. The number of vegetative cells recovered from leaves (triangles) and rocks (circles) are reported for (A) Ames (UF00738), (B) Ames-like (UF01106), (C) Vollum (UF00980), (D) Vollum-like (UF01103), and (E) Sterne (34F2), illustrating vegetative cell persistence for at least 7 dpi. Results are shown as the mean ± SEM and graphed on a log scale. Significance between days for each strain was determined by Kruskal-Wallis and Dunn’s multiple comparisons tests with leaf temporal differences identified by † = *ρ* < 0.05 and rock temporal differences identified by # = *ρ* < 0.05, ## = *ρ* < 0.01. Significance between surface types on a single day were determined by Mann-Whitney U tests with * = *ρ* < 0.05.

**Fig 4:**
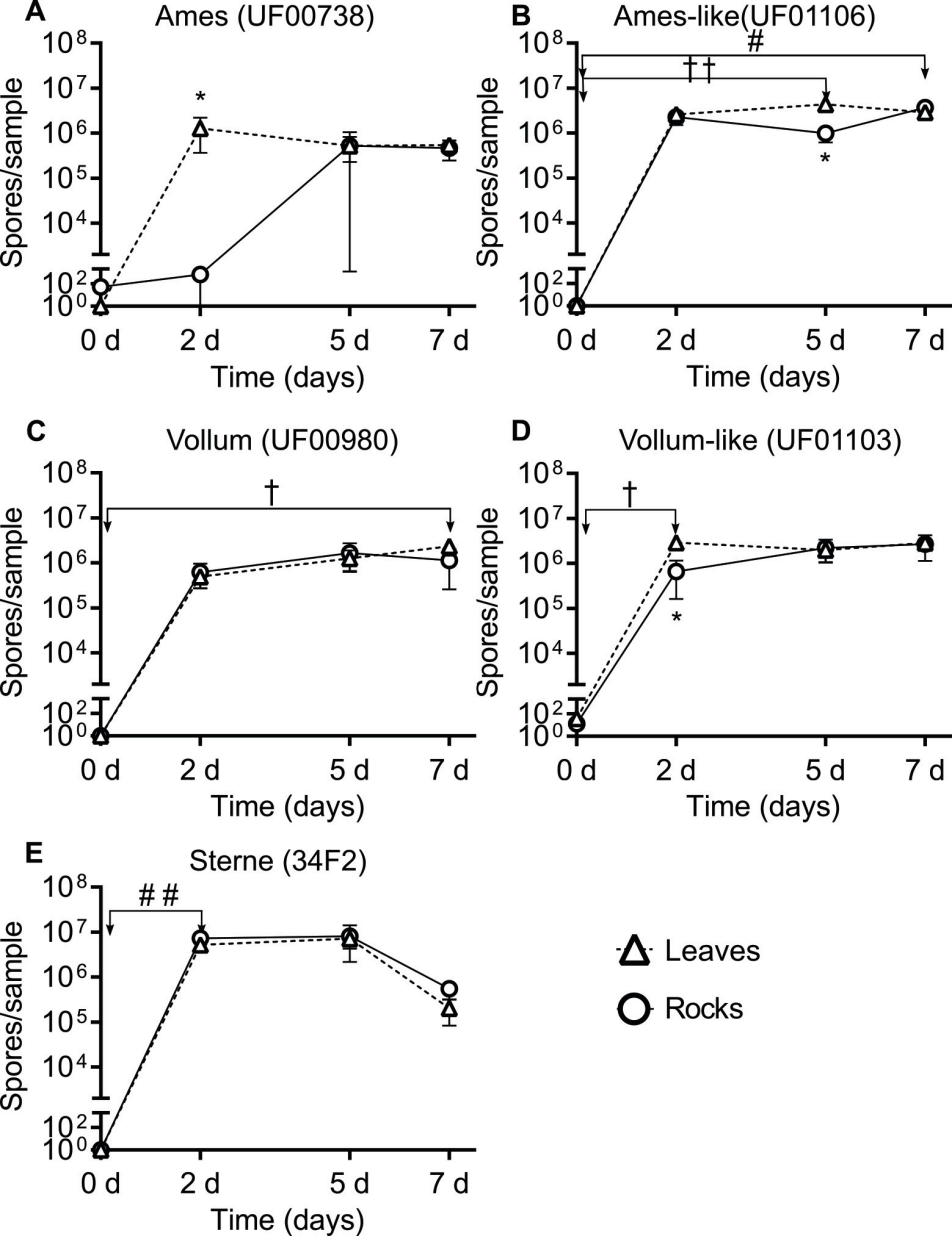
*Bacillus anthracis* spore viability on leaf and rock surfaces over a 7-day period. The number of spores recovered from leaves (triangles) and rocks (circles) are reported for (A) Ames (UF00738), (B) Ames-like (UF01106), (C) Vollum (UF00980), (D) Vollum-like (UF01103), and (E) Sterne (34F2), illustrating spore persistence for at least 7 dpi. Results are shown as the mean ± SEM and graphed on a log scale. Significance between days for each strain was determined by Kruskal-Wallis and Dunn’s multiple comparisons tests with leaf temporal differences identified by † = *ρ* < 0.05, †† = *ρ* < 0.01 and rock temporal differences identified by # = *ρ* < 0.05, ## = *ρ* < 0.01. Significance between surface types on a single day were determined by Mann-Whitney U tests with * = *ρ* < 0.05.

*Bacillus anthracis* vegetative cells and spores could persist and replicate on leaf and rock surfaces over a 7-day period. On D0, the average amount of viable vegetative cells recovered from leaves and rocks for each strain was ~10^5^ vegetative cells/leaf or rock for Ames (UF00738, Fig 3A), Ames-like (UF01106, Fig 3B), Vollum (UF00980, Fig 3C), Vollum-like (UF01103, Fig 3D), and Sterne (34F2, Fig 3E). The recovered number of bacteria from wild strains (Ames-like and Vollum-like) and laboratory-adapted strains (Ames and Vollum) increased from D0 to D2 and persisted until D7 post inoculation (Fig 3). However, it is noted that vegetative cells from Sterne were not recovered from some biological replicates during the 7-day period (Fig 3E). Although Sterne vegetative cells could replicate to the same number as fully virulent strains on both surface types on D2, Sterne showed a different temporal pattern from those strains as the total vegetative cells from Sterne gradually decreased from D2 to D7 on leaves (*ρ* < 0.05) and rocks (*ρ* < 0.01) (Fig 3E) while other strains maintained a comparatively stable amount of vegetative cells through D7. Similar to vegetative cell viability, spore concentrations from Ames (Fig 4A), Ames-like (Fig 4B), Vollum (Fig 4C), Vollum-like (Fig 4D), and Sterne (Fig 4E) strains on leaf and rock surfaces increased from an average of 10 spores/leaf or rock on D0 to 10^6^ spores/leaf or rock on D2 and persist on D5 and D7. Compared to D0, the recovered spores on the leaf surfaces were significantly increased on D2 for Vollum-like (*ρ* < 0.05, Fig 4D), D5 for Ames-like (*ρ* < 0.01, Fig 4B), and D7 for Vollum (*ρ* < 0.05, Fig 4C). On the rock surfaces, only Sterne spores increased on D2 (*ρ* < 0.01, Fig 4E) and Ames-like spores were elevated on D7 (*ρ* < 0.05, Fig 4B) when compared to D0.

By determining the differences between two surface types on multiplication and survival of *B*. *anthracis* under laboratory conditions, we observed vegetative cell densities of both the Vollum and Vollum-like strains were higher on the leaf surfaces (*ρ* = 0.0286) compared to the rock surfaces (*ρ* = 0.0286) on D7 (2.99 x 10^6^ and 2.74 x 10^6^ CFU more of each strain respectively) (Fig 3C and 3D). Likewise, the total spores recovered from leaf surfaces for the Ames (*ρ* = 0.0286) and Vollum-like (*ρ* = 0.0286) strains were higher than the total spores recovered from rock surfaces on D2 (1.28 x 10^6^ and 2.23 x 10^6^ more spores of each strain, Fig 4A and 4D respectively). On D5, the Ames-like strain sporulated on leaves better than rocks (3.36 x 10^6^ more spores and *ρ* = 0.0286, Fig 4B).

### Comparisons between the replication, persistence, and sporulation rates of Sterne and fully virulent *B*. *anthracis* strains

**Fig 5:**
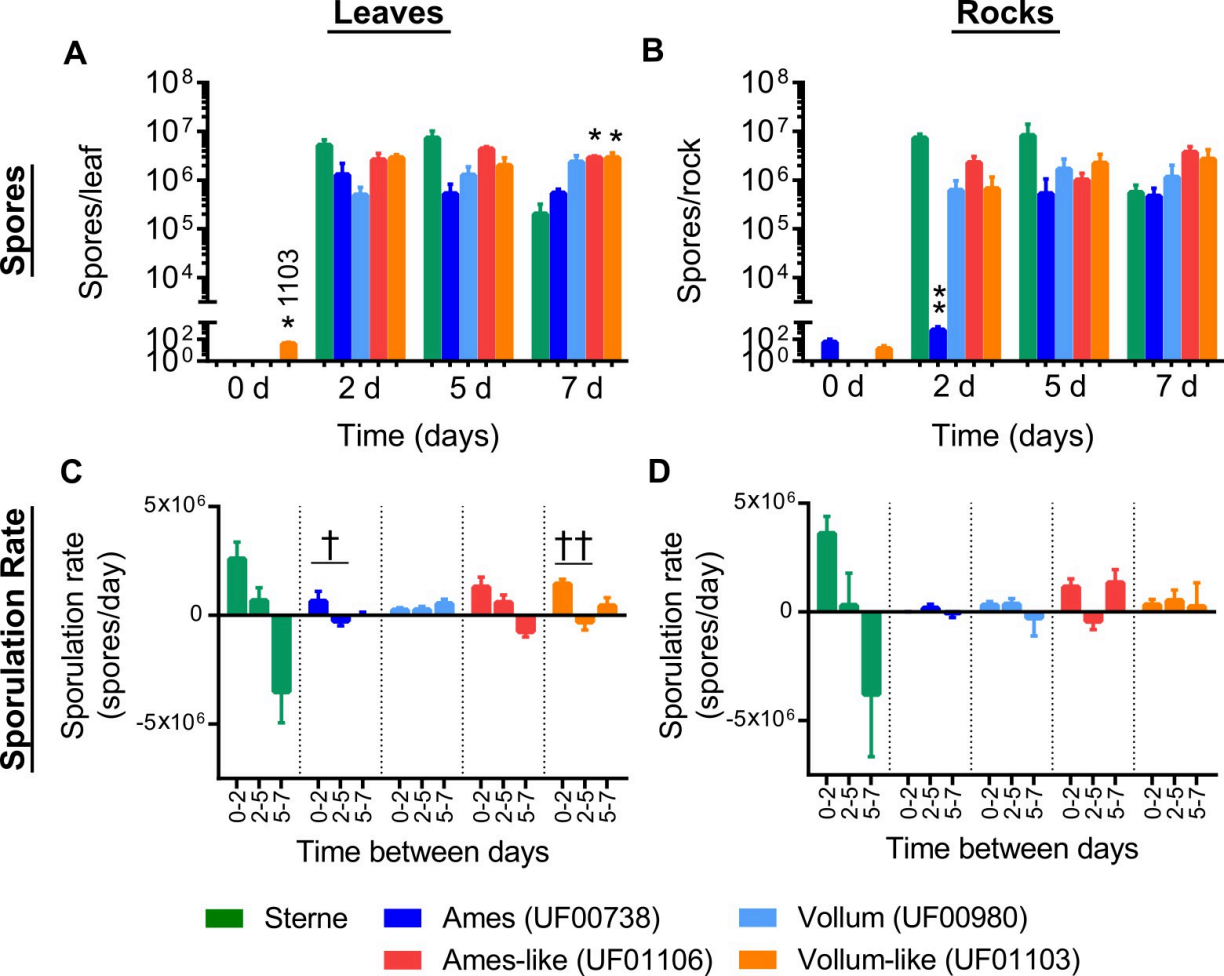
Differences in spores and sporulation rates from leaves and rocks over 7 days between Sterne strain and fully virulent strains of *B*. *anthracis*. (A) Total spores recovered from leaves, (B) total spores recovered from rocks, (C) sporulation rates on leaves, and (D) sporulation rate from rocks for the Sterne (green), Ames (UF00738, dark blue), Vollum (UF00980, light blue), Ames-like (UF01106, red) and Vollum-like (UF01103, orange) strains are presented as the mean ± SEM. Sporulation rates (spores/day) were calculated from the slope of the lines between each timepoint and graphed on a linear scale. Significant differences in total spores between the Sterne strain and all fully virulent strains were determined by Kruskal-Wallis and Dunn’s multiple comparisons tests with differences identified by * = *ρ* < 0.05, ** = *ρ* < 0.01. Significant differences in sporulation rates between days were determined by Kruskal-Wallis and Dunn’s multiple comparisons tests with leaf temporal differences identified by † = *ρ* < 0.05, †† = *ρ* < 0.01.

The pXO2 negative Sterne strain replicated and persisted on leaf and rock surfaces similarly to fully virulent strains at early time points, however at later time points we observed significantly decreased recovery of spores compared to fully virulent strains, especially from the leaf surfaces (Fig 5A). On D0, we did not detect any significant differences (*ρ* > 0.05) in the number of spores between Sterne strain and the fully virulent strains on any surfaces except from the Vollum-like strain which was found to be higher than Sterne on the leaf surface (*ρ* < 0.05, Fig 5A). As previously mentioned, there was no significant change in the number of spores from D2 to D7 for all *B*. *anthracis* strains, though the number of Sterne spores tended to drop from D5 to D7. In addition, the recovered number of Sterne spores were comparable to fully virulent strains between D2 and D7 but significantly higher than the laboratory-adapted Ames strain (*ρ* < 0.01, Fig 5B) on the rock surface on D2 and lower than fully virulent wild strains on the leaves on D7 (*ρ* < 0.05, Fig 5A). Akin to the number of spores, the recovered vegetative cell counts for the Sterne strain from leaves and rocks were not significantly different from fully virulent strains from D2 to D7 except Ames strain on the rock surface on D2 (*ρ* < 0.05, S1 Fig).

Additional differences between strains were observed in the sporulation rates between each time point (Fig 5C and 5D). Our studies demonstrated that all strains sporulated faster on leaf surfaces from D0 –D2. The sporulation rates for Ames (*ρ* < 0.05) and Vollum-like (*ρ* < 0.01) strains from D0 –D2 were significantly faster when compared to D2 –D5 (Fig 5C). Furthermore, while not statistically significant, the maximum sporulation rate was observed in Sterne strain from D0—D2 and the minimum sporulation rate was observed in Sterne strain from D5 –D7 on the leaf and rock surfaces (Fig 5C and 5D).

### Laboratory adapted vs. wild strains of *B*. *anthracis* on environmental substrates

**Fig 6:**
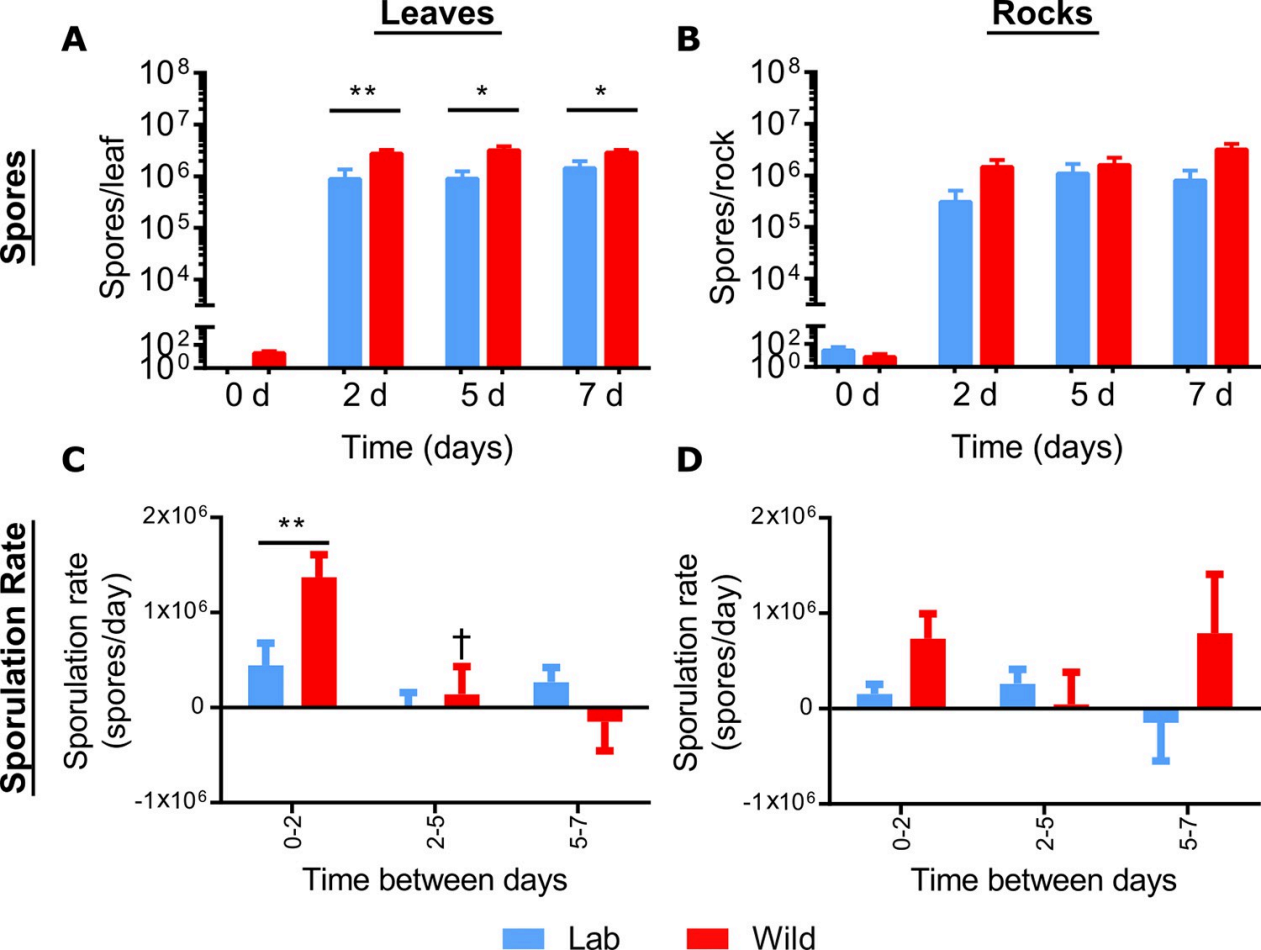
Comparing spores and sporulation rates between grouped fully virulent laboratory-adapted and wild *B*. *anthracis* strains on leaf or rock surfaces over 7 days. Grouped laboratory-adapted strains (blue) are the averaged spore titers from Ames (UF00738) and Vollum (UF00980) and grouped wild strain values (red) are the averaged spore titers from the Ames-like (UF01106) and Vollum-like (UF01103) strains. Sporulation rates (spores/day) were calculated from the slope of the lines between each timepoints and graphed on a linear scale. Data are presented as the mean ± SEM for (A) total spores recovered from leaves, (B) total spores recovered from rocks, (C) sporulation rate from leaves, and (D) sporulation rate from rocks. The significant differences between laboratory-adapted and wild strains at each time point were determined by Mann-Whitney U tests with * = *ρ* < 0.05 and ** = ρ < 0.01. Significant differences in sporulation rates between days were determined by Kruskal-Wallis and Dunn’s multiple comparisons tests with leaf temporal differences identified by † = *ρ* < 0.05.

Significantly higher wild strain spores were recovered from leaves than laboratory-adapted strains on D2 (~3-fold more; *ρ* = 0.0093), D5 (~3.5-fold more; *ρ* = 0.0101), and D7 (~2-fold more; *ρ* = 0.0281), but not rocks (Fig 6A and 6B). For laboratory-adapted and wild strains, the sporulation rates on leaves were fastest from D0 –D2, with the wild strains sporulating twice as fast as the laboratory-adapted strains (*ρ* = 0.0093, Fig 6C, Table 1). Similarly, while not statistically significant, the wild strains had a faster sporulation rate from D0—D2 on rock surfaces than the laboratory-adapted strains (Fig 6D, Table 1). On both leaf and rock surfaces, there were no significant differences in sporulation rates between the laboratory-adapted and wild strains at the later timepoints (Fig 6A and 6C, Table 1). Additionally, the vegetative cell counts were consistent between fully virulent laboratory-adapted and wild strains on both leaf and rock surfaces with average titers from both groups reaching ~10^6^ CFU/rock or leaf from D2 to D7 (S2 Fig).

**Table 1:** The comparison of sporulation rates between laboratory-adapted and wild strains at each time points on leaf and rock surfaces.

	Leaves (spores/day)			Rocks (spores/day)		
	D0-D2	D2-D5	D5-D7	D0-D2	D2-D5	D5-D7
Wild strains	1.37 x 10^6^	1.40 x 10^5^	-1.49 x 10^5^	7.33 x 10^5^	4.52 x 10^5^	7.91 x 10^5^
Lab strains	4.44 x 10^5^	2.81 x 10^3^	2.69 x 10^5^	1.54 x 10^5^	2.62 x 10^5^	-1.47 x 10^5^

### Extrapolating possible anthrax contamination levels caused by necrophagous flies at localized infectious zones

The calculated total volume of emesis spots and estimated total number of spots that can be deposited by a fly in a 24 h period are listed in S1 Table and were highly dependent on which species of fly and food source that was provided. *Chrysomya rufifacies* did not have variation in the volume of blood per spot type represented and *S*. *bullata* saw an 18 times increase in volume between translocation/tarsal tracks and emesis (both round and asymmetric blood spots).

Spot volume, number of spots from S1 Table and CFU from our laboratory experiments were used to calculate CFU per spot and CFU deposited by fly in a 24 h period depending on the food source and substrate of deposition. The estimations found that CFU contamination at 24 h would be highest on leaf surfaces contaminated by *S*. *bullata* feeding on blood. Using data from Rivers and McGregor and the methods detailed in S1 File [35], it was estimated that within 24 hours, *C*. *rufifacies* can deposit up to 2.42 μL of fluid (0.09–0.111 μL/fly spot) when provided mouse remains and as little as 0.32 μL (0.02–0.04 μL/fly spot) when provided blood (S1 Table). While *C*. *rufifacies* was the primary fly species documented to colonize deer remains, flesh flies may play a secondary role by the consumption of available blood, transferring up to 4.9 μL of fluid over a 24-h period (0.12–0.15 μL/fly spot) (S1 Table). Using our laboratory results, this equates to a single fly potentially spreading up to 3.72 x 10^4^ CFUs of *B*. *anthracis* to leaves in 24 hours (S2 Table), which by day 7 on can generate up to 2.6 x 10^4^ spores per spot and 8.62 x 10^5^ spores per day on leaves (S3 Table).

## Discussion

The stability and persistence of dormant *B*. *anthracis* spores in animal bones and soil is well documented [6, 36], however there is limited knowledge related to the pathogen’s ecology during an outbreak immediately after a host dies. The case multiplier hypothesis states necrophagous flies can distribute viable bacteria from a carcass throughout a LIZ forming a wide sphere of contamination, but research thus far has yet to investigate how long *B*. *anthracis* persists as vegetative cells or spores once transferred to environmental substrates by flies [11, 27]. Additional unknowns relate to the time frame of when a vegetative cell transitions into a spore after being deposited on environmental substrates. In the current study, we simulated necrophagous fly contamination of leaves and rocks to observe *B*. *anthracis* cell survival over 7 days with 5 different strains: the live-attenuated Sterne strain 34F2, a laboratory-adapted Ames (UF00738), laboratory-adapted Vollum (UF00980), wild Ames-like (UF01106), and wild Vollum-like (UF01103) strains. This study demonstrates that the number of vegetative cells and spores increased from D0 to D2 and persisted for at least 7 days with variations between strains, specifically, the live-attenuated Sterne strain sporulated faster than both the laboratory-adapted strains and the wild strains. A previous study sampled anthrax carcass sites in Etosha National Park and found that spores per gram of hemorrhagic blood-stained soils surrounding anthrax carcasses, whether scavenged or not, increased by several log from day 0 (death) to day 4 and kept increasing, until a week after death, to a staggering 10^8^ spores per gram of soil. Afterwards spore levels decreased [37]. This finding led Bellan et al. 2013 to question anecdotal evidence claiming *B*. *anthracis* sporulates or dies within 72 hours of exposure to air. The dynamics of bacterial replication and survival in our laboratory studies were similar in that bacterial spore quantities rapidly increased at the beginning of deposition then began plateauing towards the 1-week mark. That aforementioned study was done in Etosha National Park, Namibia and anthrax contamination beyond the carcass site was minimal. Etosha National Park is primarily grassland with few trees dominated by grazing mammals, and it is important to note that few flies were found at anthrax carcasses in this environment, minimizing the role of flies in the ecology of anthrax there [37]. The treeless environment in Etosha National Park could also reduce shade and moisture that protect viable *B*. *anthracis* as they are spread through fly emesis. We extrapolated the data from our laboratory simulation to assess the potential contamination produced via the fly transmission pathways and estimate that a single flesh-eating fly may carry as many as 6 x 10^5^ *B*. *anthracis* spores per day, further support that contaminated fly droplets may be a major component of the *B*. *anthracis* animal-to-plant transmission cycle. In a 24 h period we calculated a fly can deposit as many as 58 spots a day depending on the food source. Other work carried out in Kruger National Park, South Africa was able to isolate up to 500 spores from emesis spots by swabbing leaf and twig surfaces [38]. Swabbing is a good practice for positive and negative sampling in pathogen detection, however, it is likely to underestimate the absolute quantity of pathogen present. In our experience, whole sample bead beating in detergent is the best way to accurately enumerate pathogen levels on a sample. Given the number of emesis spots each fly can deposit per day and the numbers and methods in previous field studies, our laboratory derived numbers seem like reasonable estimates of spore contamination. That stated, our work was done in a laboratory and does not take into account many environmental variables that could reduce bacterial survival and sporulation in nature; such as UV irradiation, temperature fluctuation and humidity levels.

Here we present evidence that *B*. *anthracis* replicates and both vegetative cells and spores were able to survive on leaf and rock surfaces for at least 7 days post-inocualtion. *Bacillus anthracis* were sustained on the leaves for this period of time, this may explain why browsing species in North America, such as deer and antelope, get infected with anthrax following interactions with contaminated leaves [11, 39]. In fact, the spore concentrations on both surface types were at least 10^5^ CFU/sample or greater, constituting published infectious doses for many mammals [40, 41]. Moreover, our results demonstrated *B*. *anthracis* replicates and sporulates significantly more in the first 2–5 days after inoculation on both leaves and rocks, providing a higher chance for wildlife to get infected as early as 2 days after fly droplets are deposited on these surfaces. While it is possible nutrients from the inoculum volume sustained the proliferation of *B*. *anthracis* bacilli and increased spore pools from the initial inoculum, our results show that *B*. *anthracis* persisted on the environmental surfaces for a 7-day period which is likely beyond the extent of the nutrients included from the initial inoculum. Furthermore, a previous study discovered that experimentally exposed fly bodies still contained viable anthrax spores up to 20 days post feeding. The same study also reported viable spores recovered 6 days and 14 days post deposition from their emesis and feces, respectively [20]. These data we report here are the results of an important preliminary investigation, but longer persistence studies between 7 and 20 days, and potentially longer, are warranted to gain more knowledge about the survival time of *B*. *anthracis* on environmental surfaces.

In addition to better characterizing the survivability of *B*. *anthracis*, our investigation revealed that wild type *B*. *anthracis* vegetative cells and spores survived better on leaf surfaces than rock surfaces. Spores on leaves can amplify the number of infections during an anthrax outbreak by being ingested by browse-eating mammals, like deer or antelope, while spores on fomites, like rocks, may serve as contamination that can reseed soil with viable *B*. *anthracis* spores. Many studies show how optimal conditions are required for germination and multiplication of *B*. *anthracis* [13, 42]. For example, anthrax spores survive best in soils rich in organic matter with a high calcium level and slightly alkaline pH [13, 42–44]. Also, physical factors such as temperature and humidity were found to affect spore formation, spore persistence, and germination in *B*. *anthracis* [45, 46]. Humidity below 80% can limit bacterial germination and prolong the sporulation time [45, 46] with possible effects of this being observed in the present study where the comparatively moist environment of leaves appeared to be a more favorable surface for *B*. *anthracis* growth and replication than rocks. We also observed some rocks absorbed the bacterial inoculum faster than leaves, further implying that the contrasting moisture levels of these surfaces may be the basis for our dissimilar levels of bacterial recovery. Continued investigation into the physical properties and compositions of leaves and rocks supporting *B*. *anthracis* is needed to improve our understanding of environmental persistence of this pathogen.

To fully interpret our findings regarding *B*. *anthracis* cell persistence on leaves and rocks, we extrapolated our laboratory simulation data to estimate what may be occurring in the field when the fly transmission pathways are active. We identified *Chrysomyia rufifacies* (M.) (Diptera:Calliphoridae) and Sarcophagidae (Diptera) in the included photographs of anthrax carcasses from a 2019 West Texas outbreak (Fig 1A), where *C*. *rufifacies* was the dominant species (Fig 1B). The large abundance is not surprising as *C*. *rufifacies* is known to prefer large carrion [47] and is common in Texas during the summer [28]. Flesh flies (Diptera: Sarcophagidae) also commonly co-colonize with blow flies (Diptera: Calliphoridae), usually secondarily to blow flies [48], therefore relatively lower abundances of flesh flies at remains is expected. Adult flies attending carrion for oviposition, a protein meal for oogenesis, or mating [49] will disperse from carrion, generating fly spots in the surrounding environment. However, the size and quantity of fly spots depends on fly species and what substrate the fly consumes [35]. Since animals succumbing to anthrax expel uncoagulated blood from orifices [50], flies have direct access to soft tissues, blood, and in the event of vertebrate scavenging; organs in the abdominal cavity [51] and/or muscular tissues [52]. This suggests there can be a wide range of fly spots transported from carrion to the surrounding environment. Fly populations are known to colonize remains in multiple isolated events [53], and since anthrax can be present at the maggot and adult fly life stages [11, 22, 38], the concern of adults emerging from remains should be considered as well. For instance, a single jackrabbit carrion (*Lepus calfornicus*) can produce over 1,400 flies [54], with fly estimates increasing linearly with carrion biomass [55]. Combining the size of fly populations interacting with and reared from the deceased animal with the estimated 10^5^ spores per fly per day, we can reason that the necrophagous flies involved in the case multiplier hypothesis [11] are more than capable of depositing numerous infectious doses of *B*. *anthracis* throughout the environment surrounding infected carrion. Thusly, amplification of the LIZ diameter could occur by a few meters as suggested by fly trap placement near carcasses in Texas [11] and up to 500 m as suggested by marked fly recaptures in African rainforest anthrax zones [56].

Among the different strains of *B*. *anthracis* used here, Sterne strain sporulated more rapidly than the fully virulent strains, but Sterne vegetative cells did not persist as well by D7 when the recovered CFU significantly decreased compared to all four fully virulent strains on both leaf and rock surfaces. The sporulation rates from Ames and fully virulent wild strains were highest at early time points and gradually decreased at later time points on the leaf surface because there are less vegetative cells available to sporulate, so the sporulation rate goes down at the later time points. Interestingly, Sterne sporulates faster 2 dpi but drops rapidly at later time points on both leaves and rocks and showed distinct temporal patterns when sporulating on leaf and rock surfaces compared to fully virulent strains. It has been shown that pXO2 negative Sterne was not able to replicate or survive in blow flies under laboratory conditions [57], suggesting that *B*. *anthracis* requires the pXO2 encoded anti-phagocytic capsule for survival within blowflies and, from our data, for optimal persistence on environmental surfaces. Our findings suggest that Sterne may be used as a model for pXO2 mutant strains, but caution should be taken when comparing it to wild strains, as the mechanisms underlying how and why Sterne behaves differently from fully virulent strains are yet to be determined [58]. By comparing between fully virulent laboratory-adapted and wild strains, we observed that *B*. *anthracis* vegetative cells and spores from wild strains performed better than laboratory-adapted strains, and wild strains appeared more stable than laboratory-adapted virulent strains. Higher variability between counts with laboratory-adapted strains could be due to increased passages during which strains are known to lose genetic material that may be associated with physiological functions required for successful survival and sporulation on surfaces outside of a living host, such as leaves and rocks. These findings are consistent with a previous study in enrichment media that suggested wild strains exhibit increased growth capacity and sporulate faster because they utilize nutrients faster than laboratory-adapted strains which were slower to respond to nutrient-rich environments and exhibited prolonged lead time to sporulation [31, 32, 59]. Also, proteomic data from another study demonstrated that the genes associated with spore formation were significantly upregulated in wild strains compared to laboratory-adapted strains which is in agreement with the patterns reported here, thus we suggest that caution be taken when using highly passaged *B*. *anthracis* strains as models for wild isolates [31].

## Conclusions

Our data show the growth and persistence of viable *B*. *anthracis* on environmental substrates. We simulated the propagation of *B*. *anthracis* from carcasses by necrophagous flies onto nearby surfaces at LIZ sites during an anthrax outbreak as contaminated necrophagous flies are capable of expanding LIZ sites by moving viable bacteria from the host onto surrounding vegetation. Of the environmental substrates, leaves are the most likely source of future infection in the immediate days after host death. Continued investigations into LIZ contamination and the complexities of this disease system, like the necrophagous fly transmission pathway, will elucidate new methods to manage and prevent recurring anthrax outbreaks. Furthermore, additional studies into the impact of environmental conditions on growth and sporulation mechanisms of different *B*. *anthracis* strains will improve upon predictive modeling efforts.

## Supporting information

S1 Fig Differences in vegetative cells from leaves and rocks over 7 days between Sterne strain and fully virulent strains of *B*. *anthracis*. (A) Total vegetative cells recovered from leaves and B) Total vegetative cells recovered from rocks for the Sterne (green), Ames (UF00738, dark blue), Vollum (UF00980, light blue), Ames-like (UF01106, red) and Vollum-like (UF01103, orange) strains. The mean ± SEM were calculated and graphed on a log_10_ scale. Significant differences were determined by Kruskal-Wallis and Dunn’s multiple comparisons tests with differences between the Sterne strain and all fully virulent strains identified by * = *ρ* < 0.05.
(TIF)

S2 Fig Comparing total vegetative cells between grouped fully virulent laboratory-adapted and wild *B*. *anthracis* strains on leaf or rock surfaces over 7-day period. Grouped laboratory-adapted strains (blue) are the averaged vegetative cells from Ames (UF00738) and Vollum (UF00980) and grouped wild strain values (red) are the averaged vegetative cells from the Ames-like (UF01106) and Vollum-like (UF01103) strains. Data are presented as the mean ± SEM for (A) total vegetative cells recovered from leaves and (B) total vegetative cells recovered from rocks. The significant differences between laboratory-adapted and wild strains at each time point were determined by Mann-Whitney U tests. The data showed no significant differences between laboratory-adapted and wild *B*. *anthracis* strains at *ρ* < 0.05.
(TIF)

S1 Table Total and per spot type volume of eminence and number of spots generated by *Sarcophaga bullata* and *Chrysomya rufifacies* over a 24-hour period. Fly volume and spot calculations determined using the data published in Rivers and McGregor [35].
(XLSX)

S2 Table Estimated wild *Bacillus anthracis* (UF01106 and UF01103 strains) CFU for *Sarcophaga bullata* and *Chrysomya rufifacies*. Estimates over a 24-hour period incorporating laboratory transfer onto leaf and rock materials at Day 0 (D0). Fly volume and spot calculations determined using the data published in Rivers and McGregor [35]. Translocations and Tarsal Tracks were excluded from these estimates. CFU, colony forming units.
(XLSX)

S3 Table Estimated wild *Bacillus anthracis* (UF01106 and UF01103 strains) spores for *Sarcophaga bullata* and *Chrysomya rufifacies*. Estimates over a 24-hour period incorporating laboratory transfer onto leaf and rock materials at Day 0 (D0). Fly volume and spot calculations determined using the data published in Rivers and McGregor [35]. Translocations and Tarsal Tracks were excluded from these estimates.
(XLSX)
