# Supplementary figures and images for: Characterization of Bacillus anthracis replication and persistence on environmental substrates associated with wildlife anthrax outbreaks
Source: PLoS One. 2022 Sep 21;17(9):e0274645. doi: 10.1371/journal.pone.0274645 (PMC9491531; doi:10.1371/journal.pone.0274645)

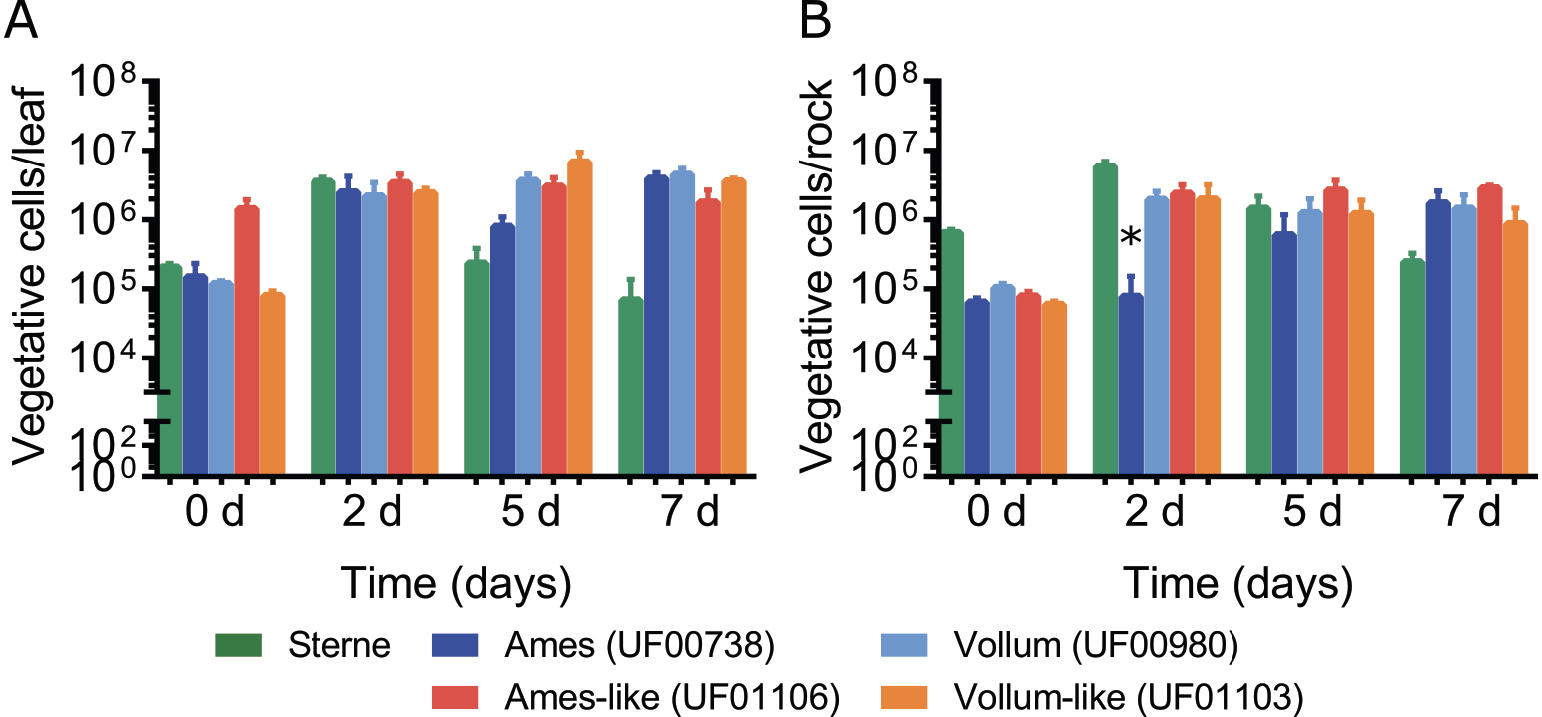

Supplement: S1 Fig — (A) Total vegetative cells recovered from leaves and B) Total vegetative cells recovered from rocks for the Sterne (green), Ames (UF00738, dark blue), Vollum (UF00980, light blue), Ames-like (UF01106, red) and Vollum-like (UF01103, orange) strains. The mean ± SEM were calculated and graphed on a log10 scale. Significant differences were determined by Kruskal-Wallis and Dunn’s multiple comparisons tests with differences between the Sterne strain and all fully virulent strains identified by * = ρ < 0.05. (TIF) [file pone.0274645.s001.tif]

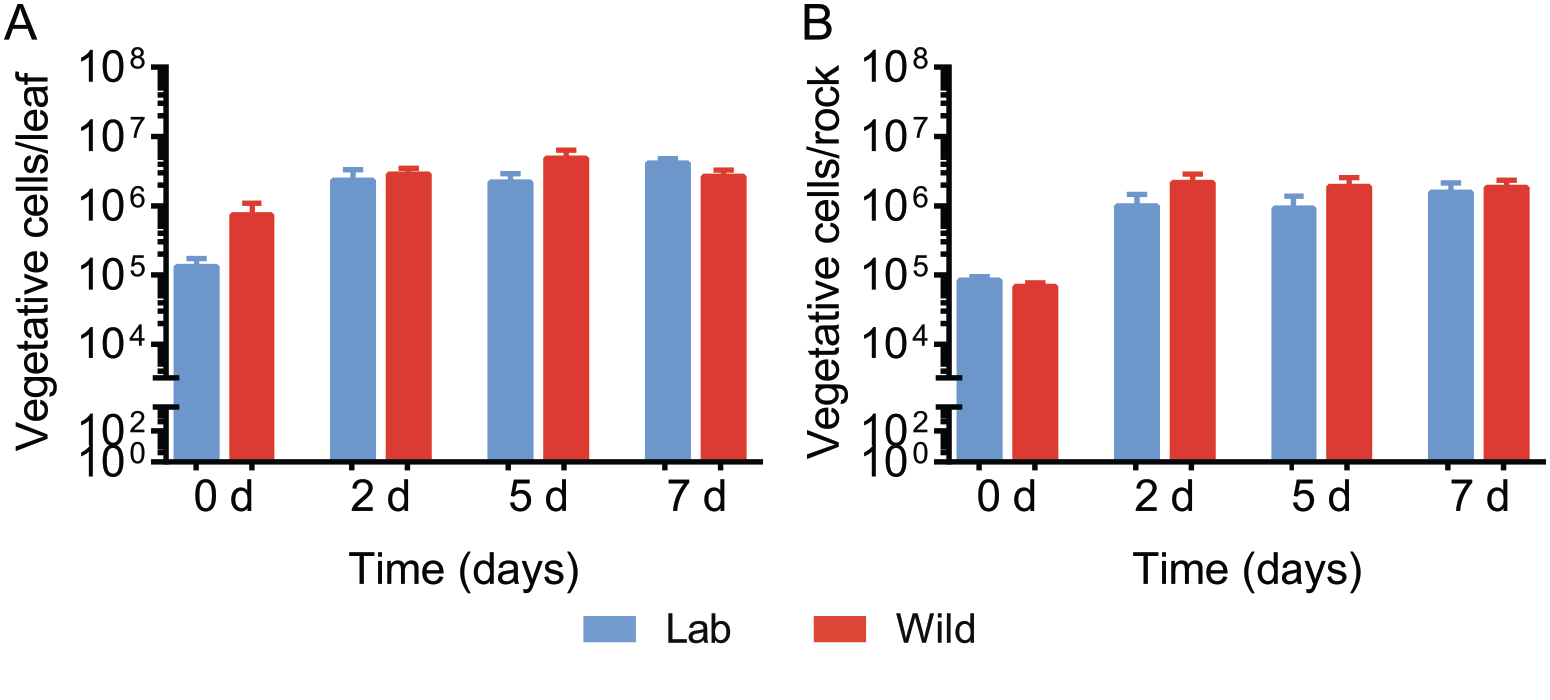

Supplement: S2 Fig — Grouped laboratory-adapted strains (blue) are the averaged vegetative cells from Ames (UF00738) and Vollum (UF00980) and grouped wild strain values (red) are the averaged vegetative cells from the Ames-like (UF01106) and Vollum-like (UF01103) strains. Data are presented as the mean ± SEM for (A) total vegetative cells recovered from leaves and (B) total vegetative cells recovered from rocks. The significant differences between laboratory-adapted and wild strains at each time point were determined by Mann-Whitney U tests. The data showed no significant differences between laboratory-adapted and wild B. anthracis strains at ρ < 0.05. (TIF) [file pone.0274645.s002.tif]
